# Quantitative analysis of medical quality intelligent management policies in China: a PMC index model approach

**DOI:** 10.3389/fpubh.2025.1716942

**Published:** 2025-11-26

**Authors:** Yanling Zhang, Kaidi Lu, Junfeng Lv, Wanping Sun

**Affiliations:** 1 Jinan University, Guangzhou, China; 2 Liaoning University of Traditional Chinese Medicine, Dalian, China; 3 Office of Science and Education, The Second People’s Hospital of Huizhou, Huizhou, Guangdong, China

**Keywords:** medical quality intelligent management policies, medical quality, sustainable advancement policies, PMC index model, quantitative analysis of policies

## Abstract

**Objective:**

This research aims to address the issues existing in the current assessment of national Medical Quality Intelligent Management policies (MQIMPs). By constructing a scientific, quantitative assessment system, it precisely identifies the strengths and weaknesses of existing policies across various aspects, providing clear direction for policy improvement, and promoting more efficient guidance of practice through intelligent management policies for medical quality.

**Methods:**

This study integrates text mining and content analysis techniques to examine the relationship between them. We construct a PMC index model. Then used the PMC index model to conduct a comprehensive assessment of the strengths and limitations of the current MQIMPs.

**Results:**

The evaluation indicates that China’s current MQIMPs system is relatively well-established, with an overall excellent performance rating. However, notable deficiencies were identified across three key dimensions: Medical Quality Control, Data Support, and Policy Audience. The relatively low scores in these areas clearly demonstrate substantial room for improvement.

**Conclusion:**

Based on the comprehensive evaluation of MQIMPs, three key recommendations are proposed. First, from the Medical Quality Control Dimension, consider adding new policies and subdividing governance areas. Second, from the Data Support perspective, establish a data lifecycle governance framework to clarify the policy core content. Third, refine audience segmentation criteria from the Policy Audience dimension. These steps will effectively develop the MQIMPs, enhancing their ability to guide practice and drive national medical quality improvement.

## 1 Introduction

Medical quality is a core pillar of the healthcare system, directly impacting public health and the sustainable development of the medical and health industry (1). Against the background of deep integration between intelligent technologies, such as big data, artificial intelligence, and the medical field, the paradigm of medical quality management is undergoing a profound transformation: it is shifting from traditional manual supervision and post-event disposal to intelligent real-time monitoring, early warning, and proactive intervention (2). Medical Quality Intelligent Management policies (MQIMPs) have emerged as key guiding documents for this transformation (3). They clarify the development direction of intelligent medical quality management, define the responsibilities of relevant stakeholders, and formulate supporting safeguards (4). Covering multiple critical links—including the development of intelligent monitoring platforms, the application of medical data standards, the training of intelligent management professionals, and the oversight of intelligent technology applications—these policies provide a systematic institutional framework for advancing the intelligent upgrading of medical quality management (5). However, the MQIMPs still face multiple challenges. On one hand, policy formulation itself has room for improvement. The alignment between policy objectives and the actual needs of intelligent technology application is not precise enough; policy tools lack diversity; and regional differences in medical and technological development levels are not fully considered (6). On the other hand, policy evaluation mechanisms are insufficient (7).

Existing evaluation methods are mostly limited to qualitative analyses or simple quantitative descriptions based on partial implementation data. These approaches cannot comprehensively and objectively assess two core dimensions: the internal consistency of policies and the completeness of policy content. As a result, it is challenging to accurately identify the root causes of ineffective policy implementation. With the accelerating pace of intelligent transformation in the medical industry, enhancing the operational effectiveness of intelligent medical quality policies has become an urgent priority. As a critical link in the policy lifecycle, policy evaluation plays a pivotal role in improving implementation outcomes.

In response to the research background and existing issues outlined above, this study uses national-level MQIMPs issued by the Chinese government in recent years as the research object. It constructs a PMC index model suitable for the quantitative evaluation of these policies. The main contributions of this study are as follows: It enriches the methodological system for evaluating intelligent medical quality policies, thereby enhancing the objectivity and accuracy of policy evaluation. It also conducts a systematic quantitative evaluation of existing MQIMPs, identifies their strengths and shortcomings, and proposes targeted pathways. This not only provides decision-making references for the formulation and revision of related policies but also promotes the high-quality development of intelligent medical quality management.

## 2 Literature review

Scholars, both domestic and international, have conducted multidimensional research on national medical quality control policies, yielding a rich body of theoretical and practical outcomes.

International research, from the perspective of policy content, international studies focus on the framework construction and core elements of medical quality control policies; for example, American scholar proposed the “Structure-Process-Outcome” model, which provides a classic analytical framework for medical quality assessment and is widely used in evaluating medical quality control policies formulated by the U. S. Agency for Healthcare Research and Quality, emphasizing the measurement of policy implementation effects from three dimensions: medical service infrastructure, service delivery processes, and final health outcomes (8). Subsequent scholars further expanded on this foundation. Some scholars put forward six goals for medical quality improvement—safety, effectiveness, patient-centeredness, timeliness, efficiency, and equity—which have become the core guiding principles for many developed countries in formulating medical quality control policies (9). Some scholars also evaluated the adequacy of current medication management in Ghana, accounting for the associated risks. The results revealed flaws in the quality prerequisites, transparency checklists, and liability mechanisms developed for AI systems, compared to existing regulations for manual processes (10). Apart from policies, some researchers have also developed integrated systems in medical management and intelligent quality management (11).

Domestic research, by contrast, closely aligns with the characteristics of China’s healthcare system to explore the evolution and implementation effects of medical quality control policies. Since the founding of the People’s Republic of China, China’s medical quality control policies have undergone a transformation from decentralized management to systematic control. Scholars have analyzed the priorities of policies at different stages by categorizing policy texts. For instance, some scholars divided China’s medical quality control policies into three phases: initial establishment, standardized development, and comprehensive deepening. Moreover, they noted that during the comprehensive deepening phase, policies place greater emphasis on multi-department collaboration and refined management (12). Meanwhile, domestic scholars also focus on issues in policy implementation (13, 14). However, existing quantitative evaluation methods have limitations: most studies focus on post-implementation effect evaluation and lack quantitative analysis of the completeness of policy texts themselves, making it challenging to identify flaws in policy design at the source (15, 16). As an emerging policy evaluation tool initially proposed by international scholars, the PMC aims to quantitatively assess the consistency, scientific validity, and feasibility of policy texts by constructing a multidimensional evaluation index system (17, 18). Its core advantage lies in converting qualitative policy elements into quantitative evaluation data; by calculating PMC index values and drawing PMC surface graphs, it can intuitively reflect the overall quality of a policy and its performance across dimensions (19). In its early stages, the PMC index was mainly applied to evaluate policies in fields such as the environment and energy. Some scholars used it to assess climate change policies across Canadian provinces, identified significant differences in goal-setting, measurement design, and implementation mechanisms, and provided clear directions for policy optimization (20). With the growing recognition of the PMC index in policy evaluation, domestic scholars have begun to incorporate it into public policy assessment, adjusting indicator systems and expanding its applications to different policy fields. In the field of health policy, some scholars have attempted to use the PMC index to evaluate medical-related policies. Some scholars constructed a PMC evaluation system with six first-level indicators—policy goals, policy measures, policy subjects, policy objects, policy guarantees, and policy timeliness—to quantitatively evaluate provincial-level hierarchical medical system policies in China, finding that these policies have overall sound quality but still need improvement in the targeting of policy measures and the completeness of policy guarantees. In evaluating cultivated land protection policies, some scholars used the PMC index to analyze China’s policies, noting that they are highly effective in reducing prices and ensuring supply but lack adequate incentive mechanisms to promote innovation and quality improvement (21).

Synthesizing the above aspects of research status, it can be seen that domestic and international scholars have conducted extensive studies in the fields of national medical quality control policies, PMC index applications, and the use of intelligent technology in medical quality control, laying a solid foundation for this study, but there are still some research gaps that highlight the necessity and innovation of this research.

First, in evaluating national medical quality control policies, existing studies mostly employ qualitative methods or single-dimensional quantitative methods, lacking a multidimensional quantitative assessment. The reproducibility of the qualitative results is poor. The same qualitative result may be interpreted differently by different people, leading to opposite conclusions. The PMC index model addresses this gap for several reasons: it transforms abstract policy text into measurable indicators, enabling precise comparisons of multiple policies across dimensions such as policy objectives, tools, and stakeholder roles. It quantifies the “degree of completeness” of policy content, avoiding the ambiguity of qualitative descriptions. It supports cross-policy and cross-regional comparative analysis, which is impossible with single-case qualitative studies. Compared to other quantitative methods, the PMC index focuses on policy text itself—a prerequisite for evaluating policy design rationality before large-scale implementation. This is particularly important for MQIMPs, as flawed policy design will directly lead to ineffective implementation, making pre-implementation text-based evaluation essential.

Second, although existing studies have introduced the PMC index into health policy evaluation, its application in medical quality control policy evaluation remains limited, and the construction of evaluation indicator systems lacks professionalism.

Third, in the collaborative research between intelligent technology and medical quality control policies, existing studies mainly focus separately on the application effects of intelligent technology and the implementation effects of medical quality control policies, lacking systematic research that combines the two; currently, no studies have analyzed the impact of intelligent technology application on medical quality control policies from the perspective of policy quantitative evaluation, nor explored how to provide support for the in-depth application of intelligent technology in medical quality control through policy optimization, resulting in a lack of effective collaborative mechanisms between intelligent technology and medical quality control policies and making it difficult to give full play to their synergistic role in improving medical quality. Based on the above research gaps, this study will construct a PMC index evaluation system for MQIMPs, identify the advantages and shortcomings of policy design through multidimensional quantitative evaluation of policy texts, and propose policy optimization paths in combination with the needs of intelligent technology application, aiming to provide theoretical support and practical reference for improving the level of intelligent medical quality control in China.

## 3 Research design

### 3.1 Data sources, samples selection and study procedures

The first stage of MQIMPs’ evolution is the Strategic Foundation Stage, marked by the release of the *Healthy China 2030 Planning Outline*. This stage proposes the “promotion of health care big data application.” Therefore, the time range for selecting policy texts in this study is set from January 1, 2016, to September 1, 2025. In this study, the relevant policies are all issued by the Chinese government. Policy texts were primarily retrieved from the official portal website of China’s central government,^1^ the Law and Regulation Database of Peking University,^2^ China National Knowledge Infrastructure,^3^ and other common retrieval platforms.

Additionally, relevant supplementary documents were searched on Baidu, Google, Bing, and other websites. The selected policy is related to the Medical intelligent control. In addition to “Intelligent Management of Medical Quality,” “Medical Quality,” “Digitalization of Medical Quality “, “Intelligence of Medical Quality “, “Medical Quality Control,” the search scope was also expanded by entering keywords such as “Medical Quality Management” to ensure a comprehensive and careful search. All policies are listed in Table 1.

**Table 1:** MQIMPs list.

Code	Policy name	Issuing agency	Date issued
P1	“Healthy China 2030” Planning Outline	CPC CC, SC	26-Aug-16
P2	Guiding Opinions on Promoting and Regulating the Application and Development of Health Care Big Data	NDRC (jointly with NHC)	24-Jun-16
P3	Measures for the Administration of Medical Quality	NHFPC	25-Sep-16
P4	Overall Plan for the Construction of Provincial Coordinated Regional National Health Information Platform	NDRC	12-Oct-17
P5	Technical Guidelines for the Construction and Application of Hospital Informatization	NHC	13-Dec-17
P6	Opinions on Promoting the Development of “Internet + Healthcare”	GOSC	28-Apr-18
P7	National Standards and Norms for Hospital Informatization Construction (Trial)	NHC	13-Apr-18
P8	Management Measures and Standards for Hierarchical Evaluation of Electronic Medical Record Application Level	NHC	3-Dec-18
P9	Measures for the Administration of National Health Care Big Data Standards, Security and Services (Trial)	NHC	12-Jul-18
P10	Notice on Further Promoting the Application of Electronic Medical Records	GONHC	22-Aug-18
P11	National Medical Security Plan for the “14th Five-Year Plan” Period	SC	23-Sep-21
P12	Measures for the Administration of Knowledge Bases and Rule Bases for Intelligent Audit and Monitoring of Medical Security Funds (Trial)	NHSA	20-Mar-22
P13	Notice on Further Promoting the Intelligent Audit and Monitoring of Medical Security Funds	NHSA	8-Sep-23
P14	Notice on Launching the Campaign for Comprehensive Improvement of Medical Quality	NHC, NATCM	1-Jun-23
P15	National Medical Quality and Safety Improvement Goals and Professional Quality Control Work Improvement Goals	NHC	2-Apr-24
P16	Medical Quality Control Indicators for 6 Specialties Including Emergency Medicine	GONHC	30-Apr-24
P17	National Medical Quality and Safety Improvement Goals	GONHC	18-Mar-25
P18	Accreditation Standards for Tertiary Hospitals	NHC	10-Jun-25
P19	Operational Manual for Performance Monitoring of National Tertiary Public Hospitals	NHC	10-Jun-25
P20	Announcement of the National Healthcare Security Administration on the Public Release of the First Batch of Rules and Knowledge Points for the “Two Databases” of Intelligent Supervision	NHSA	23-May-25
P21	Special Rectification Action Plan for Medical Quality and Safety in Medical Institutions	NHC	20-Jun-25
P22	Monitoring Indicators for the Implementation of Core Systems for Medical Quality and Safety	GONHC	3-Jun-25

SC, State Council; GOSC, General Office of the State Council; CCPCC, Central Committee of the Communist Party of China; NDRC, National Development and Reform Commission; GONHC, General Office of the National Health Commission; NHSA, National Healthcare Security Administration; NATCM, National Administration of Traditional Chinese Medicine; NHC, National Health Commission.

### 3.2 Text mining

During the policy text data processing phase, this study adopted a combination of Python tools and ROSTCM6 software to complete the entire process from raw text integration to high-frequency word extraction. The specific steps are as follows:

First, for the 22 collected MQIMPs (all in docx format), Python *docx* library was used to achieve batch reading and integration. A loop script was written to traverse all policy documents, extract text content line by line, and write it into a single text file, forming a unified policy text corpus. This process not only solved the problem of scattered storage of multiple documents but also ensured the consistency of text formatting through standardized encoding (UTF-8).

Second, the Python *jieba* library was used for word segmentation of the integrated text. Considering that policy texts contain a large number of professional terms (such as “intelligent management and control,” “electronic medical records,” and “medical insurance funds”), medical field-specific vocabulary was supplemented through the custom dictionary function before word segmentation to avoid splitting professional terms. During the segmentation process, continuous Chinese text was split into independent word units (for example, “promoting the application of healthcare big data” was split into “promoting/the application/of /healthcare/big data”). At the same time, punctuation marks, numbers, and special characters in the text were filtered out, retaining only words with actual semantic meaning.

Subsequently, stop-word removal was performed. Based on Chinese stop-word lists (the *Harbin Institute of Technology stop-word list*) and combined with the characteristics of policy texts, custom stop-words were supplemented, including meaningless auxiliary words, pronouns, adverbs, prepositions, and redundant words. Through a Python script, the word segmentation results were traversed to eliminate all words matching the stop-word list, further purifying the text data and highlighting the core semantic information of the policies.

Finally, the word list, after removing stop words, was imported into the ROSTCM6 software for the calculation of high-frequency word statistics. The software automatically calculated the frequency of each word in the policy texts through its word frequency statistics function, sorted them in descending order of frequency, and finally generated a list of high-frequency words. High-frequency words are visualized as word clouds using the *wordcloud* library in Python, which facilitates a more thorough analysis of these high-frequency words and their corresponding relationships. These high-frequency words not only reflect the core focus of the policy texts but also provide a quantitative basis for subsequent thematic analysis of policy content, enabling the study to reveal the focus and evolutionary characteristics of national medical quality intelligent management and control policies from the perspective of text semantics.

### 3.3 PMC index model construction

Based on the high-frequency word statistical results and semantic network analysis conclusions generated by the ROSTCM6 software from MQIMPs’ texts and literature data, and fully integrating the specific characteristics of the MQIMPs focused on in this study across various aspects, further targeted literature review and theoretical organization were conducted. By comparing the paradigms of variable selection in existing policy evaluation studies, indicators with low relevance to the policy attributes of this study were excluded, and characteristic dimensions suitable for intelligent control scenarios were supplemented (22). Ultimately, nine first-level variables and 27 s-level variables were identified (23, 24). In terms of weight setting, considering that the initial stage of this study aims to objectively present the coverage of each second-level indicator by policy texts, differences in the importance of different indicators were temporarily not distinguished. Therefore, the weights of all second-level variables were uniformly set to the same value. The variable values follow a [0,1] binary distribution: if the policy text explicitly mentions content related to a specific second-level indicator, the variable value is set to 1; if the policy text does not involve relevant expressions of that second-level indicator, the variable value is set to 0 (17).

The specific corresponding relationships between the above-screened first-level variables and their corresponding second-level indicators are organized. The structure of the evaluation indicators system and criteria for the second-level index are shown in Table 2, which clearly presents the hierarchical structure of the variable system. To further intuitively reflect the correlation between the “input” (i.e., indicator coverage) and “output” (i.e., the policy’s support intensity for the dimension) of policies in each indicator dimension, an input–output analysis table was finally constructed based on the identified first-level and second-level indicators. This table details the value of each second-level variable under each first-level variable, along with the corresponding policy support content. Multi-input–output table for MQIMPs is in Table 3.

**Table 2:** Structure of the evaluation indicators system and criteria for the second-level index.

First-level indicator	First-level code	Second-level indicator	Second-level code	Evaluation criteria
Policy objectives	X1	Clarity of objectives	X1-1	Whether the specific scenarios of intelligent management and control are clearly defined
		Hierarchy of objectives	X1-2	Whether the objectives are distinguished
		Forward-looking of objectives	X1-3	Whether it matches the time nodes of long-term plans
Policy entities	X2	Coverage of entities	X2-1	Whether it includes core departments such as health, medical security, and development and reform commissions
		Clarity of responsibilities	X2-2	Whether the roles of each entity in “intelligent management and control” are clarified
		Collaboration mechanism	X2-3	Whether collaborative measures such as cross-departmental data sharing and joint law enforcement are mentioned
Intelligent technology application	X3	Coverage of technology types	X3-1	Whether the specific applications of technologies such as big data, artificial intelligence, and blockchain are clarified
		Focus on application scenarios	X3-2	Whether it covers the whole process of “pre-event early warning—in-event intervention—post-event evaluation”
		Technical standards and specifications	X3-3	Whether application standards for intelligent technologies are formulated
Medical quality control dimension	X4	Clinical quality management and control	X4-1	Whether it includes professional quality control indicators
		Medical security fund management and control	X4-2	Whether intelligent fund review and monitoring rules are clarified
		Data quality management and control	X4-3	Whether the standards, security and quality of health care data are mentioned
Control measures	X5	Mandatory measures	X5-1	Whether it includes binding means such as laws, regulations, standards and assessments
		Incentive measures	X5-2	Whether incentive means such as financial support and pilot demonstrations are mentioned
		Innovative measures	X5-3	Whether new management and control methods
Data support	X6	Data platform construction	X6-1	Whether the construction requirements for data platforms at all levels are clarified
		Data sharing mechanism	X6-2	Whether the scope and process of cross-institutional and cross-regional data sharing are specified
		Data security assurance	X6-3	Whether measures such as data encryption, privacy protection and security review are included
Implementation guarantee	X7	Resource assurance	X7-1	Whether resource support such as funds, talents and technologies is mentioned
		Supervision mechanism	X7-2	Whether the supervision entity and assessment method for policy implementation are clarified
		Responsibility investigation	X7-3	Whether measures for investigating responsibilities for failure to implement policies are specified
Policy audience	X8	Coverage of audience	X8-1	Whether it includes medical institutions at all levels
		Targeting of audience	X8-2	Whether differentiated requirements are formulated for different audiences
		Guidance for audience	X8-3	Whether guidance measures such as training and publicity for the audience are included
Policy timeliness	X9	Implementation cycle	X9-1	Whether the start and end time or phase division of policy implementation is clarified
		Update frequency	X9-2	Whether the dynamic adjustment mechanism of the policy is mentioned
		Timeliness matching	X9-3	Whether the policy content matches the development of technology

**Table 3:** Multi-input–output table for MQIMPs.

First-level indicator	Second-level indicator
X1	X1-1, X1-2, X1-3
X2	X2-1, X2-2, X2-3
X3	X3-1, X3-2, X3-3
X4	X4-1, X4-2, X4-3
X5	X5-1, X5-2, X5-3
X6	X6-1, X6-2, X6-3
X7	X7-1, X7-2, X7-3
X8	X8-1, X8-2, X8-3
X9	X9-1, X9-2, X9-3

### 3.4 PMC index measure

The calculation method is as follows: First, assign values to each second-level indicator. Next, use Equations 1–3 to calculate the values of each level variable. Finally, sum all the first-level variables using Equation 4 to obtain the PMC index value for the evaluation strategy. These values are then summed up and combined with X9 to obtain the final result (25, 26).


X∼N[0,1] (1)

$$X=\{X_{R}:[0,1]\}$$ (2)

$$X_{i}(\sum_{j=1}^{n}\frac{X_{\mathrm{ij}}}{T(X_{\mathrm{ij}})})$$ (3)

$$\mathrm{PMC}=[\begin{array}{c} X_{1}(\sum_{j=1}^{3}\frac{X_{1j}}{3})+X_{2}(\sum_{j=1}^{3}\frac{X_{2j}}{3})\\ +X_{3}(\sum_{j=1}^{3}\frac{X_{3j}}{3})+X_{4}(\sum_{j=1}^{3}\frac{X_{4j}}{3})\\ +X_{5}(\sum_{j=1}^{3}\frac{X_{5j}}{3})+X_{6}(\sum_{j=1}^{3}\frac{X_{6j}}{3})\\ +X_{7}(\sum_{j=1}^{3}\frac{X_{7j}}{3})+X_{8}(\sum_{j=1}^{3}\frac{X_{8j}}{3})\\ +X_{9}(\sum_{j=1}^{3}\frac{X_{9j}}{3}) \end{array}]$$ (4)

The variable *X_i_* represents the *i*-th main variable, where *i* can take on values from 1 to 9. The notation *X_ij_* refers to the *j*-th sub-variable of the *i*-th main variable, with *j* ranging from 1 to n (27). The PMC index is calculated for 22 MQIMPs as follows: The PMC model selects nine first-order variables, resulting in a PMC index value that ranges from 0 to 9, which indicates the level of acceptability. We categorize the PMC index into six evaluation grades. A perfect policy falls within the range of 8 to 9. A superb policy is rated between 7 and just below 8. An excellent policy is rated between 6 and just below 7. A good policy falls within the range of 5 to just below 6. An acceptable policy falls within the range of 4 to just below 5. A value below 4 indicates poor acceptability and insufficient policy quality. The consistency categories for MQIMPs are detailed in Table 4.

**Table 4:** Consistency categories for MQIMPs.

PMC index interval	[0,4]	[4,5]	[5,6]	[6,7]	[7,8]	[8,9]
Policy consistency	Poor	Acceptable	Good	Excellent	Superb	Perfect

### 3.5 PMC surface construction

**Figure 1:**
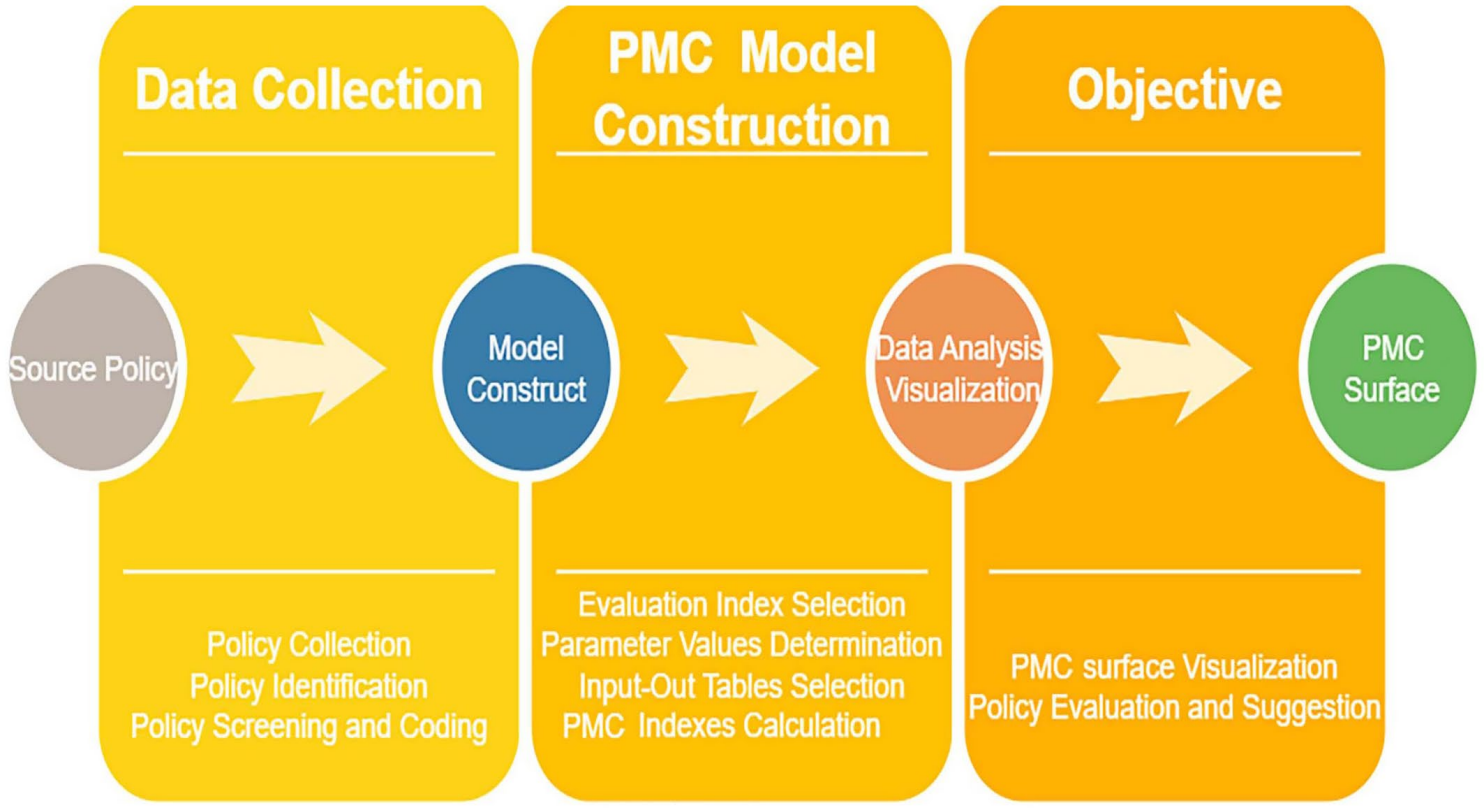
The schematic diagram of the PMC model.

To more intuitively and clearly demonstrate the strengths and weaknesses of these policies across various dimensions, the *matplotlib* library and *Axes3D* library in Python were used to construct a 3D surface plot based on the PMC index calculation results. A convex surface represents variables with higher scores, indicating a higher level of the policy in those aspects; conversely, a concave surface represents variables with lower scores, indicating a lower level of the policy in those aspects (28). The formula for PMC surface construction is as shown in Equation 5. The schematic diagram of the PMC model is shown in Figure 1.


$$\mathrm{PMC}\;\text{Surface}=[\begin{array}{c} X_{1}\;\;X_{2}\;\;\;X_{3}\\ X_{4}\;\;X_{5}\;\;\;X_{6}\\ X_{7}\;\;X_{8}\;\;\;X_{9} \end{array}]$$ (5)

## 4 Results and analysis

### 4.1 Policy scoring results and analysis

**Figure 2:**
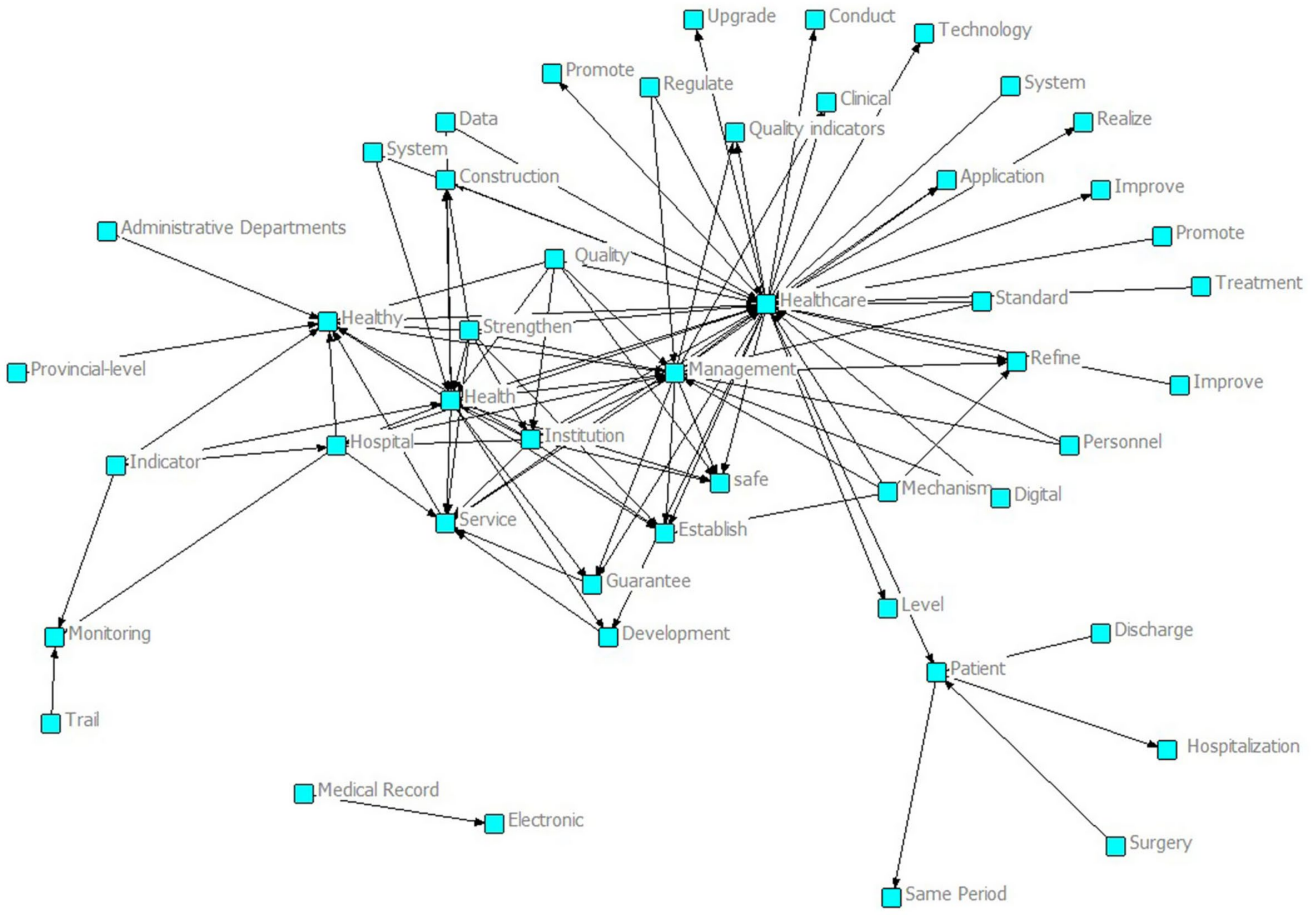
MQIMPs semantic network.

As shown in the semantic network in Figure 2, around core nodes such as “Healthcare” and “Management,” many related concepts are interconnected, including “Quality,” “System,” “Data,” “Technology,” and “Personnel.” These cover the core dimensions of intelligent management and control policies for national medical quality, including quality, systems, data, technology, and personnel. At the same time, there are words such as “Establish,” “Promote,” and “Refine” that reflect the construction and development of policies, indicating that the national intelligent management and control policies of medical quality have established a preliminary framework and have comprehensive coverage of core dimensions, providing a rich policy element foundation for subsequent quantitative evaluation and optimization path research based on the PMC index.

**Figure 3:**
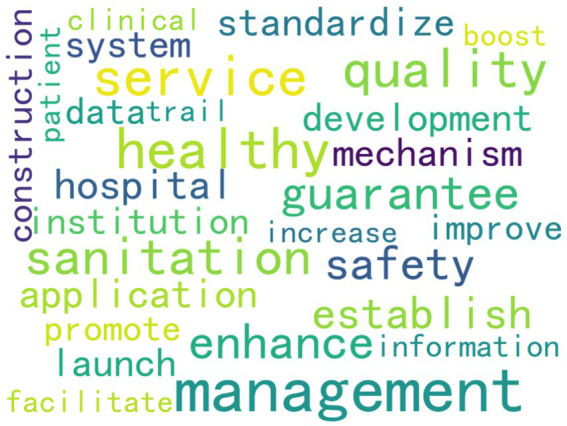
High-frequency words clouds.

As the High-frequency words shown in Table 5 and the word clouds shown in Figure 3. From these high-frequency words, we can draw the following conclusions about national medical quality, intelligent management, and control policies. Words such as “service,” “quality,” “mechanism,” “hospital,” “institution,” “safety,” “information,” and “management” indicate that the policies cover multiple aspects of medical care. They involve improving medical services, ensuring medical quality, establishing institutional mechanisms, managing medical institutions such as hospitals, ensuring medical safety, applying information technology, and overall medical management. This suggests that the policies aim to regulate and promote the medical field comprehensively. The presence of “establish,” “promote,” “enhance,” “launch,” “facilitate,” “standardize,” and “development” reflects that the policies emphasize the establishment, improvement, and promotion of medical systems and mechanisms. They strive to standardize medical practices, promote the development of the medical industry, and enhance the overall quality of medical services through institutional development and continuous improvement. Words like “safety” and “sanitation” underscore that the policies attach great importance to medical safety and sanitary conditions in medical institutions, aiming to ensure patient safety and maintain a hygienic environment. The word “information” implies that the policies also consider the application of information technology in medical quality management and control, thereby fostering the intelligent development of medical management.

**Table 5:** High-frequency words for MQIMPs.

Rank	Keywords	Frequency	Rank	Keywords	Frequency	Rank	Keywords	Frequency
1	Institution	320	11	Hospital	125	21	Promote	93
2	Management	316	12	Application	118	22	Improve	93
3	Healthy	238	13	Standardize	116	23	Construction	93
4	Service	230	14	Improve	116	24	Information	92
5	Quality	213	15	Development	113	25	Facilitate	87
6	Sanitation	199	16	System	112	26	Trail	86
7	Guarantee	174	17	Mechanism	111	27	Increase	85
8	Enhance	168	18	Launch	110	28	Patient	84
9	Safety	155	19	Institution	104	29	Boost	83
10	Establish	144	20	Data	101	30	Clinical	82

Based on the PMC index evaluation system, the first-level and second-level indicators for each policy were identified. The average PMC index of the policies is 6.29, indicating that the policies are generally Excellent. Among them, two policies are Perfect, six are Superb, eight are Excellent, two are Good, and four are Acceptable. There are no policies rated as Poor. All policy PMC index values and performance are shown in Table 6. After determining the first- level and second-level indicators using the PMC index evaluation system and calculating the index, it can be observed that the policies’ overall score is mainly in the “Superb” and “Excellent” categories. It can be clearly observed that the national medical quality intelligent management policy of our country presents the characteristics of “overall excellence, reasonable gradient, and no inefficient policies.” It not only confirms the scientific basis of policy system but also provides a clear direction for optimizing policies in the future based on their shortcomings.

**Table 6:** The PMC index values and performance of MQIMPs.

Code	X1	X2	X3	X4	X5	X6	X7	X8	X9	Sum	Rank	Performance
P11	0.67	1.00	1.00	0.67	1.00	0.67	1.00	1.00	1.00	8.00	1	Perfect
P6	1.00	1.00	1.00	1.00	1.00	1.00	0.67	0.67	0.67	8.00	2	Perfect
P2	1.00	1.00	1.00	0.33	1.00	1.00	0.67	0.67	0.67	7.33	3	Superb
P4	1.00	1.00	1.00	0.33	1.00	1.00	0.67	0.67	0.67	7.33	4	Superb
P9	1.00	1.00	1.00	0.33	0.67	1.00	1.00	0.67	0.67	7.33	5	Superb
P12	1.00	0.67	1.00	0.67	0.67	0.33	1.00	0.67	1.00	7.00	6	Superb
P13	1.00	0.67	1.00	0.67	0.67	0.33	1.00	0.67	1.00	7.00	7	Superb
P18	1.00	0.67	1.00	0.67	0.67	0.33	1.00	0.67	1.00	7.00	8	Superb
P5	0.67	0.67	1.00	0.33	1.00	1.00	1.00	0.00	1.00	6.67	9	Excellent
P8	0.67	0.67	1.00	0.67	0.67	0.67	1.00	0.33	1.00	6.67	10	Excellent
P1	1.00	0.67	0.67	0.67	0.67	0.67	0.67	0.67	0.67	6.33	11	Excellent
P14	0.67	1.00	0.67	0.67	1.00	0.00	1.00	0.67	0.67	6.33	12	Excellent
P7	0.67	0.67	1.00	0.33	0.67	1.00	1.00	0.00	1.00	6.33	13	Excellent
P20	1.00	0.33	1.00	0.67	0.67	0.33	0.67	0.67	1.00	6.33	14	Excellent
P21	0.67	1.00	0.67	0.67	0.67	0.00	1.00	0.67	1.00	6.33	15	Excellent
P19	0.67	0.67	1.00	0.67	0.33	0.33	0.67	0.67	1.00	6.00	16	Excellent
P10	0.67	0.67	1.00	0.67	0.33	0.33	0.67	0.33	1.00	5.67	17	Good
P15	0.67	0.67	0.33	0.33	0.33	0.00	1.00	0.67	1.00	5.00	18	Good
P3	0.67	0.67	0.33	0.33	0.33	0.00	1.00	0.67	0.67	4.67	19	Acceptable
P16	0.33	0.67	0.33	0.33	0.33	0.00	0.67	0.67	1.00	4.33	20	Acceptable
P17	0.33	0.67	0.33	0.33	0.33	0.00	0.67	0.67	1.00	4.33	21	Acceptable
P22	0.33	0.67	0.33	0.33	0.33	0.00	0.67	0.67	1.00	4.33	22	Acceptable
Average	0.76	0.76	0.80	0.53	0.65	0.45	0.85	0.59	0.89	6.29	NA	Excellent

### 4.2 PMC surface results and analysis

**Figure 4:**
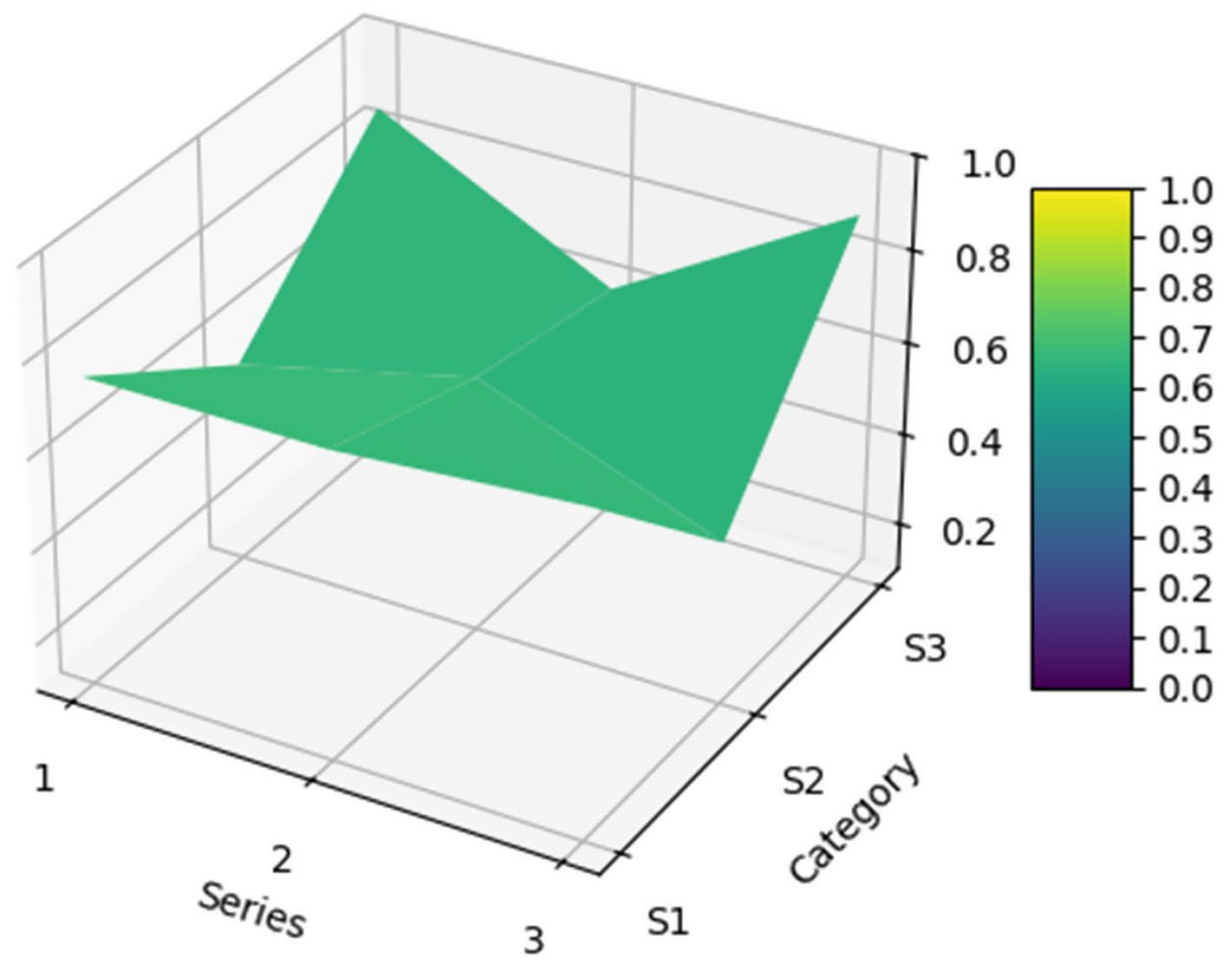
MQIMPs average PMC surface.

**Figure 5:**
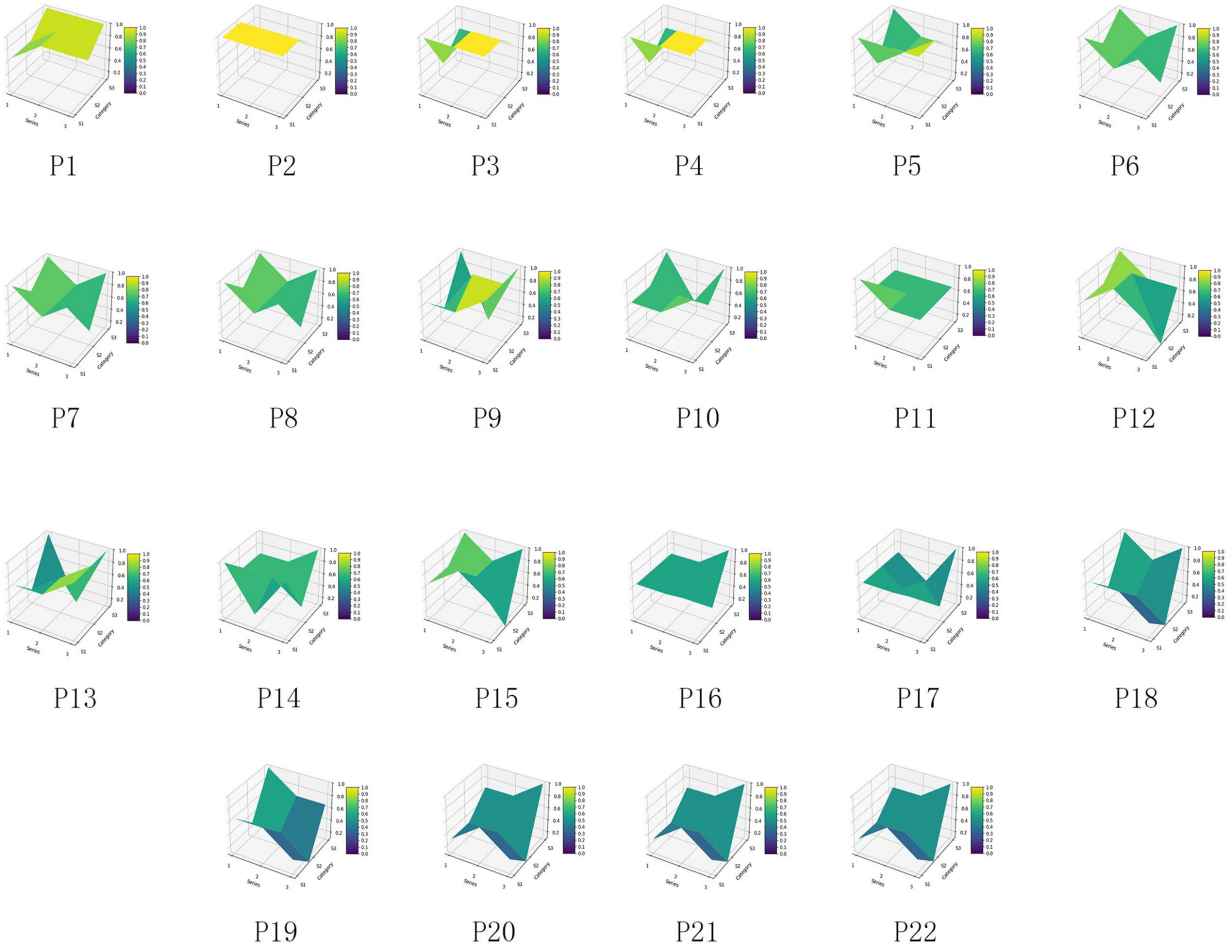
All MQIMPs PMC surfaces.

For the 22 policies, a PMC was created based on the scores of the first-level variables. Additionally, as shown in Figure 4, an overall PMC surface was created based on the average scores of each primary variable. Moreover, as shown in Figure 5, all MQIMPs PMC surfaces have been created. Among all the policies, the highest-ranked were Policy P11 and Policy P6. Although these two policies had the same total score, their performance differed in intensity across dimensions: their scores ranged from 0.67 to 1.00. Specifically, Policy P11 outperformed Policy P6 in dimensions X7 (implementation guarantee), X8 (policy target audience), and X9 (policy timeliness), while Policy P6 excelled in dimensions X1 (policy objective), X4 (medical quality control dimension), and X6 (data support). Among the 22 policies, 14 received the highest evaluation grade of “Superb.” Policy P5 received a relatively high overall evaluation. However, its score in dimension X8 (policy target audience) was 0, suggesting that even high-scoring policies may have weaknesses. Two other policies (P10 and P15) received the evaluation grade of “Good,” with P15’s score in dimension X6 (data support) being 0. Furthermore, four policies received the evaluation grade of “Acceptable,” and the score of the X6 (data support) dimension for all these four policies was 0, which also indicates that among all the primary variables, X6 (data support) is the dimension with the poorest performance in the overall policy evaluation.

### 4.3 Discussion on the scores of the primary variables

**Figure 6:**
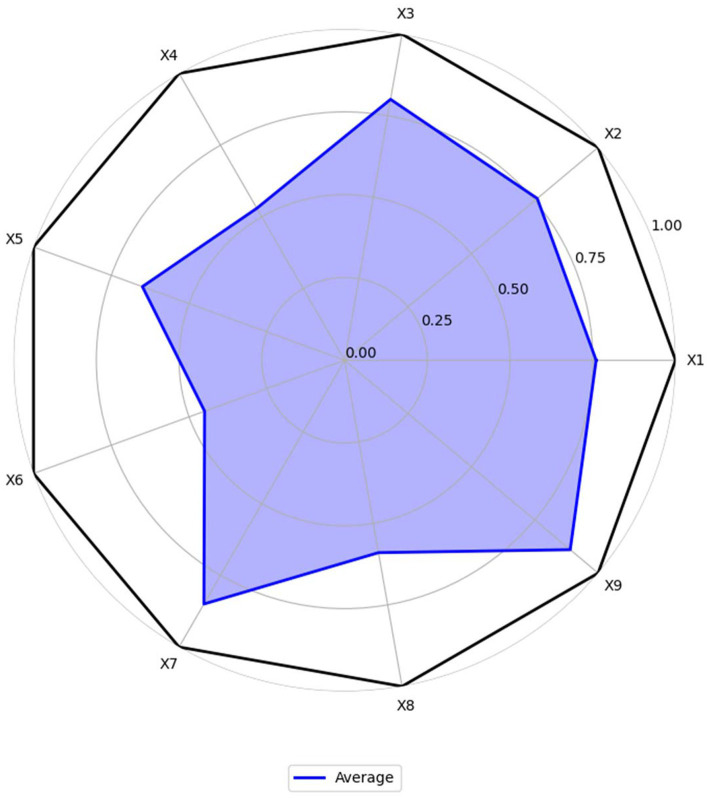
Radar chart for MQIMPs.

Based on the average scores, make the radar chart for MQIMPs. As shown in Figure 6, six of the nine first-level variables have scores above 0.6. In contrast, 3 have scores below 0.6, namely X4 (medical quality control dimension), X6 (data support), and X8 (policy audience). The following provides a detailed analysis of the score situations of all primary variables.

#### 4.3.1 Policy objectives (X1)

According to the assessment results, the average score for the policy objectives dimension is approximately 0.76, indicating a relatively high overall rating. Among them, P2, P4, and P6 have core goals of “improving the quality of medical care for the entire population and establishing an intelligent control system,” and these goals are highly consistent with the “Healthy China” strategy and the development trend, with a score of 1.00. In contrast, P16, P17, and P22, although they have specific local goals of “standardizing medical quality” and “refining professional indicators,” do not fully align with the overall orientation of “intelligent control,” and the synergy between the goals and the policy system is insufficient, with a score of 0.33. This indicates that the top-level policy objectives for intelligent medical quality control in China have been unified. However, some policies focused on specific fields remain unclear in their objectives and could be further strengthened to better align with the goals and the system.

#### 4.3.2 Policy entities (X2)

The average score of the policy entities dimension is approximately 0.76, with a relatively high overall rating, reflecting that the medical quality intelligent control in China has established a “government-led, multi-department collaboration, and institution implementation” entity system. P9, P11 clearly define the rights and responsibilities of multiple entities such as the National Health Commission, the Medical Insurance Bureau, and local governments, forming a “top-level coordination—middle-level execution—grassroots implementation” division of labor mechanism, with a score of 1.00; P20 mentions multiple entities but does not detail the collaborative processes among departments (such as the division of responsibilities in data sharing and the cross-departmental linkage mechanism in intelligent monitoring), with a score of 0.33. It can be seen that a framework for multi-entity participation has been established, and the collaborative rights and responsibilities can be further clarified.

#### 4.3.3 Intelligent technology application (X3)

The average score for the intelligent technology application dimension is 0.80, with a high overall rating, reflecting the high attention China’s medical quality control policies are paying to “intelligent” technologies. P12, P13, and P18 have stable scores of 1.00 in this dimension. At the same time, P3, P15, and the other four policies recognize the importance of technology but do not specifically explain its application path, with a score of 0.33. This indicates that although the policy level has a clear technical orientation and has established the core idea of “technology-driven control,” it can further shift from “macro guidance” to “micro implementation,” clarify the operational standards and implementation steps of technology application, and promote the deep expansion of application scenarios.

#### 4.3.4 Medical quality control dimension (X4)

The average score for the medical quality control dimension is approximately 0.53, indicating a relatively low overall rating and reflecting the imbalance in the policy’s coverage of the “control dimension.” Only the “Opinions on Promoting the Development of ‘Internet + Medical Health’” (P6) has a “Perfect” level policy that comprehensively covers core dimensions such as “medical service quality, medical safety, medical insurance fund compliance, and data security,” with a score of 1.00; all other policies have scores ranging from 0.33 to 0.67. The policy coverage of core dimensions is relatively comprehensive; however, specific fields still require supplementation, and there is considerable room for improvement.

#### 4.3.5 Control measures (X5)

The average score for the control measures dimension is approximately 0.65, indicating a medium overall rating, with a notable feature of “complete framework, details to be optimized.” P2, P4, P6, and P11 have scores of 1.00 in this dimension, while P10 and P19 have scores of 0.33. This indicates that the policy system is basically complete. However, there is a lack of differentiation and flexibility, and further efforts are needed to enhance the “differentiation” and “dynamicity” of the measures.

#### 4.3.6 Data support (X6)

The average score of the data support dimension is approximately 0.45, indicating a low overall score. Although some policies (such as P2, P4, P9) have a score of 1.00, others (such as P3, P15, P16) have a score of 0, significantly lowering the average score for this indicator. These policies with low scores did not mention the connection between “data support” and “intelligent control.” A consensus on the importance of data has been reached, but the data governance mechanism still needs improvement. Special policies should be adopted to address data governance gaps.

#### 4.3.7 Implementation guarantee (X7)

The average score for the implementation guarantees dimension is approximately 0.85, and the overall score is high, ranking as the highest among the nine indicators. Policies primarily provide guarantees in the “organization, resources, and supervision” areas. All policies have a score between 0.67 and 1.00 in this dimension. The guaranteed framework is essentially in place, and the resources and oversight efforts are also relatively mature.

#### 4.3.8 Policy audience (X8)

The average score for the policy audience dimension is approximately 0.59, indicating a relatively low overall score. The policies cover the core audiences, such as “government departments, medical institutions, and medical insurance institutions,” but there is insufficient attention to “indirect audiences.” For example, P7 has a score of 0 in this dimension, and the policy does not mention the audience group. Another example is P11; although the overall score is high, there is still room for improvement in the audience group score. The core audience coverage is comprehensive; however, the indirect audience still needs to be expanded.

#### 4.3.9 Policy timeliness (X9)

The average score for the policy timeliness dimension is approximately 0.89, and the overall score is high, with all policies scoring between 0.67 and 1.00 in this dimension. The policy time nodes are clear, and the dynamic adjustment mechanism is also relatively mature and complete.

### 4.4 Discussion on the PMC surface results

According to all the PMC diagrams, China’s national medical quality intelligent management policies are generally in a good situation. From the radar coverage of the 22 PMC diagrams, there is no “full-dimensional low-quality” policy in which all nine first-level indicators have scores below 0.5. The radar charts for all policies have achieved a score of 0.67 or higher on at least three indicators. This feature indicates that China’s national medical quality intelligent management policy system has basic effectiveness, and that all policies are playing their respective roles. It also demonstrates that the policy design at the local level is well thought out and grounded in science. The logic behind this phenomenon is that the formulation of China’s medical quality intelligent management policies has always been guided by the “Healthy China” strategy and the medical development plan as the top-level guidance. Before the policy is issued, it needs to undergo multi-department research and expert argumentation to ensure that the policy has feasibility in key dimensions such as “core goals, implementation framework, and time nodes,” avoiding the occurrence of “no goals, no subjects, no measures” “three-no policies.” Therefore, based on the overall performance of PMC diagrams, all medical quality intelligent management policies in China can play a “basic control role” in specific fields, with no “ineffective policies” or “formal policies,” providing stable policy support for the practice of medical quality intelligent management. Analyzing the PMC index scores of policies from both domestic and international perspectives can help improve the policies.

From a domestic perspective, the MQIMPs can be further strengthened. For the low-scoring variables X4, X6, and X8, analyzing the improvement directions for these variables can better enable policymakers to update, add to, or reformulate policies. The Medical Quality Control Dimension (X4) is low, indicating that the authority of a specific executive department is limited and the effectiveness of multi-departmental collaborative work is poor. In such cases, it is advisable to establish a mechanism to enhance collaboration among multiple departments. Data Support (X6) is low, indicating an unreasonable resource allocation. Consider increasing or modifying the current mechanism to achieve a more reasonable allocation of resources. Policy Audience (X8) is low, indicating incomplete policy coverage. Therefore, more suggestions or opinions need to be collected to update the current system.

From an international perspective, the U. S. medical quality management system is characterized by decentralized governance, with standards and supervision mainly led by private organizations and market mechanisms. At the same time, federal and state governments play a supplementary regulatory role. This model fosters flexibility in policy response to market demands. However, it also leads to inconsistencies across regions—some states lack clear frameworks for intelligent medical quality supervision, resulting in fragmented application of AI tools in clinical quality control. In contrast, China’s policy system, guided by the “Healthy China” strategy, exhibits strong top-level coordination. The uniform emphasis on “core goals” and “implementation frameworks” across national and local policies ensures that intelligent management tools are promoted in a coordinated manner. The EU has taken a rigorous regulatory approach to intelligent medical quality, most notably through the 2024 Artificial Intelligence Act, which classifies medical AI as high-risk and mandates full-lifecycle quality oversight, including transparency requirements for algorithms and clinical validation. This focus on risk prevention ensures high safety standards. However, it can lead to lengthy approval cycles for policy implementation, with some member states delaying the rollout of intelligent quality management tools due to overly strict compliance requirements. China’s policies strike a balance between risk control and practical effectiveness. As indicated by the PMC indicators, policies maintain strong performance in “feasibility” and “time nodes” while incorporating basic risk safeguards. This balanced model offers insights for the other regions seeking to accelerate the adoption of intelligent technologies without sacrificing regulatory rigor.

### 4.5 PMC model stability and accuracy analysis

Based on the PMC index model, the previous study conducted a quantitative evaluation of 22 MQIMPs in China from 2016 to 2025. The results showed that the overall performance of these policies reached the “Excellent” level (with an average PMC index of 6.29). However, the first three-level indicators scored significantly lower than others, namely the Medical Quality Control Dimension (X4, average score 0.53), Data Support (X6, average score 0.45), and Policy Audience (X8, average score 0.59), which have become the core directions for policy optimization. To further verify the stability and accuracy of the PMC index evaluation results, we conducted additional analysis.

For stability, we use the sensitivity analysis method. With this method we can first to verify the reliability of the interpretation of low scores, confirm whether the low scores of X4, X6, and X8 are caused by indicator setting deviations rather than actual defects of the policies themselves; second to locate key sensitive indicators, identify the secondary indicators that contribute the most to the fluctuation of the PMC index, providing more precise targets for policy optimization; third to enhance the persuasiveness of the model, verify the scientific and anti-interference ability of the previous conclusions by simulating result differences under indicator changes. In combination with the previous evaluation results, analysis objects were selected from 9 first-level indicators and 27 secondary indicators, prioritizing low scores and assigning high weights. For first-level sensitive indicators, focus on the three dimensions with the lowest scores: X4 (Medical Quality Control), X6 (Data Support), and X8 (Policy Audience). For secondary sensitive indicators with a coverage rate of 36.36%; X6-1 and X6-2 (Data Platform Construction and Data Sharing Mechanism, both only eight policies mentioned this indicator, with a coverage rate of 36.36%, making it the lowest-scoring indicator in the X6 dimension); X8-3 (Audience Targeting, only one policy mentioned this indicator, with a coverage rate of 4.55%, which is the lowest score in the both 27 dimensions). Given the binary nature of policy text evaluation, the coverage status of secondary indicators was graded, and three types of Scenarios for Sensitivity Analysis were explicitly specified in Table 7. As shown in Table 8, the final scores for the three types of variation scenarios ranged from 6 to 7, indicating that the PMC results after processing the disturbance term remain Excellent. This shows that the PMC model is highly stable in reaching conclusions about the direction of MQIMPs.

**Table 7:** Three types scenario for sensitivity analysis.

Type	Operation definition	Simulation logic
Baseline scenario	Keep the original scores unchanged	Serve as a control benchmark for sensitivity analysis
Partial optimization scenario	Increase the policy coverage rate of the target secondary indicator by 30%	Simulate the effect of policies supplementing key content on a small scale
Full optimization scenario	Increase the policy coverage rate of the target secondary indicator to 100%	Simulate the ideal effect of policies fully filling the gap of this indicator

**Table 8:** The final scores for the three types scenario for sensitivity analysis.

Sensitive indicator	Baseline scenario	Partial optimization scenario	Full optimization scenario	Evaluation change or not
X4-2	6.29	6.32	6.50	No
X6-1	6.29	6.32	6.50	No
X6-2	6.29	6.32	6.50	No
X8-3	6.29	6.30	6.61	No

For accuracy, a Pearson chi-square test was performed on the variables X1-X9. As the Pearson chi-square test Results shown in Table 9, the results indicated that the *p*-values for X4, X6, and X8 were greater than 0.05, whereas those for the other first-level variables were less than 0.05. It indicates that for X4, X6, and X8, there is a weak quantitative correlation among the second-level indicators within each of these three first-level variables. This suggests that none of these three indicators has a particularly prominent second-level indicator, indicating that they are either consistently low or consistently high. Based on the PMC results, it was found that these three indicators are precisely the ones below the average value. From a statistical perspective, the PMC model constructed from the first- and second-level indicators in this study demonstrated high accuracy.

**Table 9:** Pearson chi-square test results.

First-level code	Second-level code	Mean	Within SD	Pearson Chi-2	*p*-value
X1	X1-1	0.955	0.213	16.995	<0.001
	X1-2	0.864	0.351		
	X1-3	0.455	0.51		
X2	X2-1	0.955	0.213	34.815	<0.001
	X2-2	1	0		
	X2-3	0.318	0.477		
X3	X3-1	0.727	0.456	8.238	0.0162
	X3-2	1	0		
	X3-3	0.682	0.477		
X4	X4-1	0.636	0.492	3.771	0.1517
	X4-2	0.364	0.492		
	X4-3	0.591	0.503		
X5	X5-1	0.955	0.213	16.950	<0.001
	X5-2	0.364	0.492		
	X5-3	0.636	0.492		
X6	X6-1	0.364	0.492	4.4	0.1108
	X6-2	0.364	0.492		
	X6-3	0.636	0.492		
X7	X7-1	0.727	0.456	6.6	0.0369
	X7-2	1	0		
	X7-3	0.818	0.395		
X8	X8-1	1.565	3.603	40.9914	1.2555
	X8-2	1.739	3.991		
	X8-3	0.087	0.288		
X9	X9-1	0.682	0.477	15.6610	<0.001
	X9-2	1	0		
	X9-3	1	0		

Although the model’s stability and accuracy have been verified, it still exhibits some subjectivity. Specifically, the selection of primary and secondary indicators is still primarily done by humans, with other quantitative analysis methods in policy studies used as a supplement. If the secondary indicators in the model cannot fully replace the primary variables, the results obtained may not be remarkably accurate. For example, the score of the first-level indicator in this article is aggregated from 3 secondary indicators. Suppose these three secondary indicators cannot fully replace the score of the first-level indicator. In that case—that is, if a larger variable influences the first-level indicator’s score—the scores obtained in this article will appear less accurate. Therefore, future research on this policy can also delve deeper into the selection of primary variables and the determination of secondary indicators, thereby obtaining a more in-depth analysis and suggestions for the MQIMPs.

## 5 Conclusions, recommendations and implications

### 5.1 Conclusion

This study conducted an assessment and analysis of the consistency of the PMC index of MQIMPs from 2016 to 2025 through text mining. We identified the key characteristics of these policies and found that although they were constantly improving, they still had certain limitations. We examined the PMC values of each second-level indicator separately and proposed suggestions accordingly. This study is the first quantitative examination of the consistency of MQIMPs following their implementation in China, thereby filling a gap in the literature. The results revealed that the average PMC index for MQIMPs across the 22 items was 6.29, indicating that the policies are generally Excellent. According to average PMC diagrams,

Moreover, as the Evaluation Result of MQIMPs shown in Table 10, all the policies were classified into four categories: 2 are Perfect, six are Superb, eight are Excellent, two are Good, and four are Acceptable. Among the MQIMPs, the average values of the three primary variables—X4, X6, and X8—were relatively low.

**Table 10:** Evaluation result of MQIMPs.

Performance	Poor	Acceptable	Good	Excellent	Superb	Perfect
Policy numbers	0	4	2	8	6	2

To further enhance the practicality of policy recommendations and address the identified limitations, concrete improvement paths and clear priorities are proposed based on the low average values of primary variables X4, X6, and X8, as well as the PMC index classification results. First, we can establish a cross-departmental coordination mechanism to standardize data sharing across relevant agencies and set up quarterly joint supervision meetings to resolve implementation bottlenecks promptly. Second, we can formulate a phased funding support plan, allocating a certain percentage of special funds to pilot regions with weak infrastructure first, and evaluating the effectiveness of the funds used every 6 months to optimize resource allocation. Third, we can develop a dynamic assessment system for policy beneficiaries, incorporating feedback from enterprises and the public into the policy revision process, and updating assessment indicators annually to align with actual development needs. By implementing these specific improvement measures, we can strengthen our policies.

### 5.2 Recommendations

#### 5.2.1 Improvement for the medical quality control dimension

Consider adding new policies and subdividing areas. New policies could include a policy similar to “Classification Guide for Medical Quality Intelligent Control Dimensions,” which clearly retains existing content such as “diagnostic norms, medical record quality, medical safety (such as surgical complication rate), and compliance with medical insurance funds (such as over-treatment control).” This ensures the policy’s compatibility with the traditional medical quality system. At the same time, introduce an intelligent dimension, clearly including “Internet medical diagnosis quality (such as online consultation misdiagnosis rate, prescription compliance), remote medical security (such as equipment operation norms, cross-institutional data transmission security), intelligent monitoring algorithm quality (such as fund intelligent review accuracy, AI-assisted diagnosis error rate), and data privacy protection (such as patient information de-identification standards)” for emerging dimensions, filling the gap in intelligent scenario control. Subdivided areas can be categorized by “medical scenarios”: for specialized fields such as emergency, pediatrics, and psychiatry, as well as institution types such as grassroots medical institutions and internet hospitals, formulate differentiated control dimension details. For example, for the emergency field, based on the existing “emergency rescue efficiency” dimension, add “emergency intelligent triage accuracy” and “emergency data real-time transmission integrity” as intelligent-related dimensions; for grassroots medical institutions, simplify the “complex medical technology quality dimension” and strengthen “intelligent auxiliary diagnosis application quality” and “interconnection quality between grassroots and superior hospitals” as adaptable dimensions.

Specific Operational Steps can be as follows four steps. First, establish a cross-departmental drafting team. The National Health Commission takes the lead, and members include experts from the Medical Quality Control Centre, the Internet Medical Supervision Department, the Medical Insurance Bureau, and technical teams from artificial intelligence enterprises (e.g., enterprises engaged in medical AI algorithm development). The team is responsible for researching existing policies and intelligent medical scenarios. Second, conduct a scenario-based demand survey. The team distributes questionnaires to 50 representative medical institutions (including 10 tertiary hospitals, 20 grassroots medical institutions, and 20 internet hospitals) to collect pain points in intelligent quality control. Third, draft the “Classification Guide for Medical Quality Intelligent Control Dimensions.” Based on the survey results, the guide clearly retains traditional dimensions (diagnostic norms, medical record quality, etc.) and adds detailed standards for intelligent dimensions. Last, publicly solicit opinions and revise. The draft guide is published on the NHC’s official website for a 30-day public comment period, and revisions are made based on feedback from medical institutions, experts, and the public before final issuance.

#### 5.2.2 Improvement for data support

A data lifecycle governance framework can be established to clarify the policy’s core content. Specifically, the policy must require the mandatory inclusion of data governance elements: All policies related to intelligent medical quality control must clearly cover the four core elements: “data standards, data sharing, data security, and data quality.” Data standards should unify medical data coding and intelligent monitoring data formats to ensure consistency and interoperability across all systems. The National Health Commission should lead the formulation of the “Medical Quality Intelligent Control Data Standard Manual,” and in the policy, it is necessary to clearly state “must comply with the XX standards in the manual”; The scope and process of data sharing across departments (health, medical insurance, and drug supervision) and across institutions (hospitals, third-party testing institutions, and internet medical platforms) should be clearly defined. For example, “The medical insurance department can obtain the non-compliant medical treatment data from the hospital’s intelligent monitoring, but a data confidentiality agreement must be signed, and it can only be used for fund supervision”; Data Security should Tiered protection measures for data should be stipulated (such as patient privacy data as “core level,” encrypted storage is required; medical treatment statistics data as “ordinary level,” de-identification sharing is allowed), and the accountability mechanism for data leakage should be clearly defined; A data quality process should be established, for example, “The data uploaded by the hospital to the regional health information platform must undergo integrity verification and accuracy verification before it can be used for intelligent control.” Specific Operational Steps can be divided into the following 4 phases. The first phase is the formulation of Data Standards. The first step is to compile the “Medical Quality Intelligent Control Data Standard Manual.” The manual unifies medical data coding and intelligent monitoring data formats, with specific requirements for both. Then issue mandatory implementation requirements. In the policy, clearly state: “All medical institutions and internet medical platforms must comply with the data coding and format standards in the ‘Medical Quality Intelligent Control Data Standard Manual’ from 1 January of the following year. Non-compliant institutions will be suspended from using the national intelligent quality control platform until rectification is completed.”​ The second phase is the standardization of Data Sharing (about 6 months, co-led by the NHC, the Medical Insurance Bureau, and the State Drug Administration). Define the scope and process of cross-departmental and cross-institutional sharing. Formulate a “Medical Data Sharing Management Detailed Rules.” Then establish a sharing supervision platform. The NHC builds a national medical data-sharing supervision platform to record each department and institution’s sharing behavior (e.g., time of sharing, data scope, purpose of use). Once abnormal sharing (e.g., using data for commercial purposes) is found, the platform will automatically issue an early warning and notify the supervision department. The third phase is implementation of Tiered Data Protection (about 4 months, led by the NHC and Ministry of Public Security). Clarify data classification standards. Divide medical data into two levels with specific protection measures. Then establish a mechanism for accountability for data leakage. Clearly define the responsibilities of relevant parties in the policy. The last phase is the establishment of the Data Quality Process (about 3 months, led by the NHC and local health commissions). Formulate a full-process quality verification standard. Require medical institutions to implement the following verification steps before uploading data to the regional health information platform: integrity verification and accuracy verification. Then establish a data quality evaluation mechanism. The regional health information platform scores the data quality of each institution quarterly (with integrity and accuracy accounting for 50% each). Institutions with a score ≥90 are rated “excellent” and given priority in policy support (e.g., increasing the quota for remote consultation); institutions with a score <80 are rated “unqualified” and required to rectify within 1 month. If rectification fails, the institution’s access to the intelligent quality control platform is restricted.

#### 5.2.3 Improvement for policy audience

Refine the audience segmentation criteria. First, the core audience should be retained and further strengthened, which includes government departments (health and wellness, medical insurance, and cyber information departments, responsible for policy formulation and supervision), medical institutions (hospitals and grassroots health institutions, responsible for policy implementation), and medical insurance institutions (responsible for intelligent fund review and monitoring). It is essential to clearly specify their “specific responsibilities” (e.g., “Medical insurance institutions need to provide feedback on abnormal data from intelligent fund monitoring to the health and wellness department every month”). Second, new indirect audiences can be added, including medical information technology service providers (such as electronic medical record system developers and AI medical equipment providers, responsible for offering technical support for intelligent control) and medical associations (such as hospital associations and physician associations, responsible for policy training and industry self-discipline), while clearly defining their technical responsibilities and supporting responsibilities. Finally, new stakeholders can also be incorporated, including patients (policy beneficiaries, responsible for providing feedback on policy implementation effects) and third-party evaluation institutions (responsible for conducting independent assessments of policy implementation effects), and their “participation channels” should be clearly specified (e.g., “Patients can report unreasonable issues in intelligent control through the intelligent feedback platform of medical institutions, and such feedback should be responded to within three working days”).

### 5.3 Implications

Based on this research, the findings provide valuable insights for optimizing the structural design of intelligent management policies for national medical quality. Moreover, it establishes a reusable framework for policy optimization at both theoretical and practical levels. From a domestic perspective, the national medical quality intelligent management policies are a key means for the central government to support the sustainable development of national medical quality. The effectiveness of these policies directly depends on the scientific combination of their elements and the accuracy of information transmission. The shortcomings identified in this study offer clear avenues for improvement for policymakers. From an international perspective, the policy optimization framework proposed in this study has global applicability for other countries seeking to promote the sustainability of their traditional medicine or cultural heritage. Many countries around the world face the common challenge of balancing the protection of traditional Medical knowledge with the promotion of modern innovation. The significance of this research lies not only in promoting the sustainable development of traditional Chinese Medical Quality in China, but also in providing valuable references for other countries that wish to protect and develop their own traditional Medical Quality and modern innovation.

## Data Availability

The original contributions presented in the study are included in the article/supplementary material, further inquiries can be directed to the corresponding author.

## Footnotes

1
https://www.gov.cn/

2
https://www.pkulaw.com/

3
https://www.cnki.net/
